# Fighting the SARS-CoV-2 Pandemic: Focusing a New Lens on COVID-19

**DOI:** 10.34133/2022/9879646

**Published:** 2022-07-21

**Authors:** Fudi Wang

**Affiliations:** ^1^School of Public Health, Zhejiang University School of Medicine, Hangzhou 310058, China; ^2^Hengyang Medical School, University of South of China, Hengyang 421001, China

Recent statistics regarding COVID-19, the disease caused by the SARS-CoV-2 virus, show that the COVID-19 pandemic has had a major global impact, with the virus infecting more than 3.5 billion individuals and causing more than 15 million deaths since the start of the pandemic [1, 2]. Back in April 2020, when the World Health Organization (WHO) declared COVID-19 a global pandemic, we performed the first systematic review and meta-analysis and found that patients with a preexisting chronic condition such as hypertension, cardiovascular disease, chronic kidney disease, or diabetes had a significantly higher risk of developing severe COVID-19 and/or COVID-19-related death [3]. Today, many countries have returned to some degree of normalcy, loosening many of their COVID-19 restrictions; however, the threat of new waves of infection due to emerging variants of SARS-CoV-2 remains. It is therefore imperative that we continue to identify new aspects of COVID-19 and the underlying virus. In this special edition of *Research*, we present new findings regarding COVID-19, including seven original research articles and one perspective of our current understanding of this highly contagious viral disease.

The Omicron variant of SARS-CoV-2 spread extremely rapidly, quickly reaching more than 100 countries and regions, resulting in numerous waves of COVID-19 around the world [4, 5]. Understanding the structure of key proteins in the Omicron variant is therefore essential for the development of the corresponding vaccines and pharmaceutical interventions [6, 7]. To this end, Jumper and colleagues obtained extremely accurate structures of the spike (S), membrane, and nucleocapsid proteins expressed by the Omicron variant using DeepMind's AlphaFold2 algorithm [6, 8, 9]. Their analysis revealed that several mutations in the S protein's receptor-binding domain (RBD) may affect the interaction between the RBD and angiotensin-converting enzyme 2 (ACE2). The structure of the S1 N-terminal domain in the Omicron variant differs significantly from the original SARS-CoV-2 strain, reducing the variant's recognition by antibodies and resulting in the potential for immune escape and a decreased efficacy of existing vaccines. Interestingly, however, the conserved RBD epitope recognized by the recombinant monoclonal antibody S309 is not changed in the Omicron variant, indicating that the S309 antibody may still be effective for the treatment of patients infected with the Omicron variant of SARS-CoV-2 [10]. The study by Shi and colleagues demonstrates the value of simulating high-precision structural data using the AlphaFold2 algorithm, an approach that can be used to rapidly determine key structures in other variants, providing first-hand information to help fight the ongoing global pandemic. In this respect, their work establishes a robust structural framework for studying newly emerging strains and variants of SARS-CoV-2.

The development of biomimetic experimental platforms that can recapitulate the pathogenic features associated with SARS-CoV-2 infection provides a valuable set of tools to complement conventional research models. For example, the so-called “organ-on-a-chip” model is an emerging *in vitro* cell culture technology that can mimic—with high precision—the microarchitecture and microenvironment of human organs in an artificial microfluidic device. Because this platform can mimic both physiological and pathophysiological features at the organ level, it is considered a promising new strategy for advancing the field of virology [11]. Cao and colleagues recently developed a biomimetic lung-on-a-chip to recapitulate the alveolar and vascular physiology and pathophysiology for use as an innovative new model for studying viral infection [12]. The authors then studied the potential effects of mechanical forces applied to the chip, examined the tissue's inflammatory response to the immunostimulant polyinosinic-polycytidylic acid (poly(I:C)), and investigated the entry process of a SARS-CoV-2 pseudovirus in order to mimic SARS-CoV-2-induced pathogenesis. This biomimetic alveolus-on-a-chip provides a new *in vitro* platform for studying alveolar physiology and pathophysiology and may serve as a powerful new tool for studying viral infection in human pulmonary tissue.

Interestingly, Chen and colleagues report in this issue of *Research* that SARS-CoV-2 infection can also affect several sperm parameters commonly used to indicate male reproductive health [13]. In their study, the authors conducted a meta-analysis of 14 studies in order to determine differences in sperm parameters between 521 patients with COVID-19 and 653 uninfected controls and found differences in several parameters, particularly total sperm motility and sperm concentration. In addition, they found that the semen quality was significantly decreased 3 months after SARS-CoV-2 infection and then tended to recover due to the production of new spermatozoa following a cycle of spermatogenesis. Notably, this meta-analysis indicates that testicular function should be monitored closely in male patients with COVID-19 and early interventions may be warranted when abnormalities are detected.

Recently, Maimaitiyiming et al. reported that mild heat treatment can promote the ubiquitin-mediated proteolysis of the SARS-CoV-2 RNA-dependent RNA polymerase (also known as nonstructural protein 12 or NSP12) [14]. With respect to the underlying mechanism, mild heat stress rapidly induces the ubiquitination of NSP12 by the ubiquitin E3 ligase ZNF598, followed by proteolysis via the ubiquitin-proteasome pathway. The authors showed that applying prolonged heat treatment to SARS-CoV-2-infected Vero E6 cells (a cell line established from monkey kidney epithelial cells) significantly reduced both the viral RNA load and the virus titer [14]. Notably, they also found that mild heat treatment had a similar effect on the P323L mutant form of NSP12, a mutation identified in several variants of SARS-CoV-2, including the Omicron variant [15]. Thus, by taking advantage of the thermal sensitivity of both wild-type NSP12 and the P323L mutant form of NSP12, it may be possible to develop heat-based interventions and reduce or prevent COVID-19 outbreaks—including the current wave caused by the Omicron variant—particularly in regions without adequate access to medical supplies or more conventional interventions.

Despite the growing level of vaccine-induced immunity around the world, emerging SARS-CoV-2 variants continue to cause recurrent waves of infection and disease. To date, 87.8% of China's population has been immunized against SARS-CoV-2; however, a key question is whether this population is “immunologically prepared” for future waves [16]. It is therefore essential that we understand the current state of comprehensive immune protection against the SARS-CoV-2 variants present in various Chinese populations. To address this question, Li and colleagues examined specific humoral and cellular immunity against SARS-CoV-2 variants in several groups, including individuals who had been exposed to specific variants and/or received vaccines and booster shots [17]. The results consistently revealed reduced titers of neutralizing antibodies against SARS-CoV-2 variants; however, they found that additional exposure to antigen—regardless of whether this was due to a breakthrough infection or a booster vaccine—restored or improved the capacity to neutralize variants, including the Omicron variant. Furthermore, they found that vaccinated individuals retained their T cell-mediated immunity against the Omicron variant, presumably compensating for reduced neutralization; as a result, long-term adaptive immunity may serve as a key factor protecting against SARS-CoV-2 variants and even new coronaviruses that may emerge in the future. These data also indicate that a higher humoral immune response may induce a stronger cellular response. Taken together, these findings provide a rationale for the current booster vaccination strategy, as administering a vaccine to increase immunity is far safer than viral infection, particularly among individuals with chronic illness. In addition, children and adolescents can have a less effective humoral response compared to adults and even the elderly; thus, special attention should be paid to this age category, particularly as our knowledge grows with respect to how comorbidity may be associated with more severe COVID-19 in this vulnerable patient population [3].

Yang and colleagues used peptide microarrays to map the linear peptide epitopes (LPEs) that can be recognized following SARS-CoV-2 infection and/or vaccination and found extensive overlap between LPEs recognized by IgMs produced by nonhuman primates and patients [18]. Notably, these LPEs were localized to functionally relevant virus regions and were aligned with reported binding sites for neutralizing antibodies. The authors also found vaccine-specific LPEs that were mapped to sites either known to be affected or likely to be affected by structural changes induced by proline substitutions introduced into the S protein encoded by mRNA-based vaccines. These important findings highlight the importance of mapping LPEs to known functional regions, thus providing a platform to identify new targets for therapeutic interventions.

There is also an urgent need to develop additional diagnostic tools to further analyze the disease state. Nanobodies have been shown to play a major role in the treatment of disease, even as a therapeutic intervention for use in COVID-19. The conventional methods for evaluating the neutralizing efficacy of nanobodies against the RBD use techniques such as primarily biolayer interferometry (BLI) and the plaque reduction neutralization test (PRNT); however, these methods are relatively expensive, laborious, time-consuming, and invasive. To overcome these issues, Liu and colleagues developed a straightforward and quantitative method to dynamically evaluate the infection pathway of SARS-CoV-2 [19]. Specifically, the authors developed ^68^Ga-Nb1159, a nanobody-based radiotracer that can be used to visualize the location and distribution of the SARS-CoV-2 RBD with a high target-to-background ratio; in addition, they demonstrated a potential therapeutic response due to blocking the interaction between ACE2 expressed in the host cell and the virus' RBD. Given its relative ease of use, this new method will likely be useful for evaluating other neutralizing nanobodies designed to target the SARS-CoV-2 RBD and to determine the precise location of the RBD in the body, thereby guiding precision therapy.

In summary, significant progress has been made—and continues to be made—with respect to our understanding of the SARS-CoV-2 virus and COVID-19. Although the SARS-CoV-2 vaccines developed to date have been shown to be safe and effective, saving countless lives and allowing many countries to return to nearly prepandemic conditions, vaccination does not provide full protection to everyone. As new variants emerge and give rise to new waves of infection and disease, expanding our understanding of this disease remains an essential task. Finally, to aid in our global fight against COVID-19, multidisciplinary collaborations will play a key role in the rational design of novel therapeutic interventions.
